# Silicone Rubber Triboelectric Nanogenerator for Self-Powered Wide-Range Frequency Vibration Monitoring

**DOI:** 10.3390/nano16070420

**Published:** 2026-03-30

**Authors:** Lei Guo, Hong Zeng, Junqi Li, Juntian Liu, Yongjiu Zou, Jundong Zhang

**Affiliations:** College of Marine Engineering, Dalian Maritime University, Dalian 116026, China; engineering_glay@dlmu.edu.cn (L.G.); zenghong@dlmu.edu.cn (H.Z.); lijunqi@dlmu.edu.cn (J.L.); ljt1208@dlmu.edu.cn (J.L.); zouyj0421@dlmu.edu.cn (Y.Z.)

**Keywords:** vibration sensor, triboelectric nanogenerators, machinery condition monitoring

## Abstract

With the advancement of automation and intelligent manufacturing, mechanical vibration monitoring has become crucial for equipment health assessment. This study proposes a triboelectric nanogenerator (TENG)-based vibration sensor featuring a silicone rubber composite structure. The sensor comprises a silicone rubber layer sandwiched between polyethylene terephthalate (PET) films backed by conductive fabric electrodes, all supported on a polylactic acid (PLA) arch frame. Through systematic structural optimization, the device employing Dragon Skin-30 silicone (1 mm thickness) and conductive fabric electrodes achieved a significant enhancement in output voltage and superior sensitivity compared to initial designs. The optimized sensor operates over a broad detection range for acceleration (5–50 m/s^2^), amplitude (0.1–2 mm), and frequency (1–300 Hz), and exhibits high linearity (R^2^ ≥ 0.97974) in acceleration sensing. Quantitative comparison with existing triboelectric nanogenerator (TENG) vibration sensors confirms that the proposed SR-TENG outperforms most reported devices in terms of comprehensive detection range and linear sensing performance. Durability tests over 2 h confirmed stable output without degradation. Practical validation on marine blower equipment demonstrated accurate frequency monitoring, closely matching actual vibration characteristics. This work presents a novel approach to self-powered vibration sensing and supports the development of intelligent, sustainable industrial monitoring systems.

## 1. Introduction

In an era of rapid technological development and profound industrial transformation, the precise monitoring of mechanical vibrations is indispensable across numerous fields [1]. The need spans from tracking the operational status of large-scale machinery in industrial production to monitoring vibrations associated with human physiological activities [2,3], driving the demand for highly sensitive and reliable mechanical vibration sensors [4]. Although conventional sensors can meet essential detection requirements in standard environments, their limitations become pronounced with technological advances and increasingly complex application scenarios. A primary constraint is their reliance on external power supplies [5], which severely restricts deployment in extreme or remote locations where stable power is inaccessible, such as in wilderness exploration or deep-sea operations [6]. Furthermore, their performance and operational longevity are susceptible to environmental variations, including fluctuations in temperature and humidity, as well as interference from strong electromagnetic fields [7,8], often resulting in signal distortion and compromised long-term stability. Consequently, the development of a self-powered, highly sensitive, environmentally robust, and sustainable mechanical vibration sensing technology has become particularly urgent [9,10].

In recent years, triboelectric nanogenerators (TENGs) have emerged as a promising technology for self-powered sensing, leveraging the coupled effects of contact electrification and electrostatic induction [11,12]. Among various materials for constructing TENGs, silicone rubber is particularly suitable for high-performance devices owing to its excellent flexibility, elasticity, and favorable dielectric properties [13]. When paired with conductive fabric electrodes—which also offer good flexibility and conductivity—the constraints on silicone rubber motion typically imposed by rigid metal electrodes (e.g., copper or aluminum) are effectively alleviated, ensuring stable sensor operation even under complex mechanical deformation [14].

However, current TENG configurations using silicone rubber as the triboelectric material are predominantly designed for energy harvesting [15], typically employing thin silicone layers with large surface areas. Moreover, silicone rubber is often utilized merely as an encapsulating material in many TENG devices [16]. Consequently, vibration sensors that utilize smaller-area silicone rubber as the primary functional component remain in an early stage of development, presenting several unmet design challenges. Key issues include optimizing the thickness and hardness of the silicone rubber to balance mechanical durability with high vibration sensitivity, and minimizing the constraint on silicone rubber motion imposed by the electrode’s flexibility, as these factors directly govern the sensor’s sensitivity [17]. Substantial research is further required to ensure the sensor’s stability and reliability under harsh operational environments—such as exposure to high temperature, humidity, and intense vibration—to guarantee long-term performance consistency [18]. Beyond intrinsic performance, a significant challenge lies in the practical integration of such sensors into diverse existing monitoring systems, necessitating seamless compatibility with various platforms and data interfaces.

In response to the aforementioned challenges, this study proposes a high performance triboelectric nanogenerator mechanical vibration sensor based on a silicone rubber, designated as the SR-TENG. The sensor features an innovative arch-frame structure, wherein a silicone rubber is sandwiched between two PET-conductive fabric layers to form a freestanding vibration unit, which is then supported by two arched PLA frames. The working mechanism of this silicone-rubber-based TENG is thoroughly analyzed. A systematic investigation is conducted to evaluate its performance under complex operating conditions, encompassing variations in frequency, amplitude, and acceleration, with comprehensive assessment of key metrics including sensitivity, linearity, and long-term stability. Practical application demonstrations, such as monitoring shipboard blowers, are presented to validate its effectiveness in real-world scenarios. This work aims to address the existing technological gaps, provide new momentum for the development of mechanical vibration sensing technology, and promote its broader application across various fields, thereby contributing to the intelligent transformation and sustainable development of modern industry.

## 2. Materials and Methods

### 2.1. Fabrication of the SR-TENG

The SR-TENG was fabricated through a precise layer-by-layer assembly process with strict dimensional control. Two arch-shaped support frames were first produced from PLA via 3D printing. The frames exhibited outer dimensions of 40 mm in length, 30 mm in width, and 8 mm in height, with an inner cavity measuring 32 mm by 22 mm. After printing, the frames were cleaned with isopropyl alcohol (IPA). The active triboelectric layer was a sheet of silicone rubber prepared from Dragon Skin-30 (Smooth-On, Inc., Easton, PA, USA). It had a thickness of 1 mm, a length of 34 mm, and a width of 24 mm. It was fabricated by mixing the base and curing agent at a 10:1 weight ratio, followed by degassing, casting, and thermal curing at 60 °C for 2 h. Flexible electrodes were prepared by laminating conductive fabric onto PET films. Each electrode measured 36 mm by 26 mm. Copper wires were attached to the conductive fabric using silver paste, and the assembly was subsequently cured at 80 °C for 30 min. The silicone rubber was carefully aligned and sandwiched between two such electrodes to form the vibration layer. This integrated layer was then secured between the two PLA frames using epoxy resin. Finally, all exposed copper leads were insulated with heat-shrink tubing. The entire fabrication procedure was performed in a Class 1000 cleanroom environment.

### 2.2. Electrical Measurement

The SR-TENG was mounted on an electrodynamic shaker (JZK-20) and was driven by amplified sine waves from a function generator (YE1311) and then an amplifier (YE5852). The acceleration was measured by a commercial accelerometer (KS96.100) and displayed in DASP software (Version V10, Inelta, Chengdu, China). The electric output signals, including open-circuit voltage, short-circuit current, and transferred charge, were measured by an electrometer (Keithley 6514).

For temperature and humidity performance verification, the SR-TENG was mounted on the electrodynamic shaker and placed in a programmable temperature and humidity test chamber. Tests were carried out strictly according to the CCS specifications for marine electromechanical equipment. For temperature tests, five temperature gradients were set: −25 °C, 0 °C, 25 °C, 50 °C, and 70 °C. The sensor was stabilized at each temperature for 2 h before testing to ensure consistent material properties. For humidity tests, five relative humidity gradients were set at a constant 25 °C: 20% RH, 40% RH, 60% RH, 80% RH, and 95% RH. The sensor was stabilized at each humidity level for 1 h before testing.

## 3. Results and Discussion

### 3.1. Structure and Working Principle of SR-TENG

**Figure 1:**
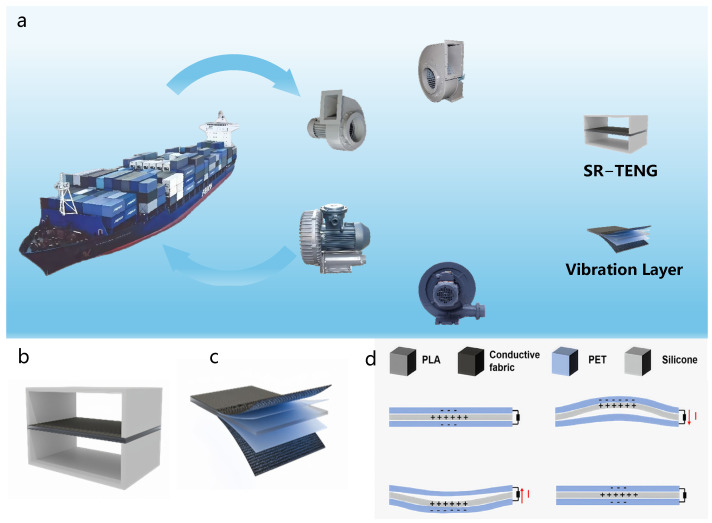
Application scenario, structure and working principle of SR-TENG. (**a**) Schematic diagram of the application scenarios of the SR-TENG for shipboard mechanical vibration monitoring. (**b**) Overall structure of the SR-TENG. (**c**) Layered structure of the main vibration layer. (**d**) Working principle of the SR-TENG based on the coupling of triboelectrification and electrostatic induction, where the red arrows indicate the direction of electron transfer during the contact-separation process.

Figure 1a illustrates the application of the SR-TENG in mechanical vibration monitoring, with a focus on condition monitoring for critical equipment in marine engineering and industrial manufacturing. The sensor can be directly mounted onto key equipment surfaces, such as marine blowers and air compressors, and is also adaptable to various rotating or reciprocating machinery on production lines for capturing real-time operational vibration signals. As shown in Figure 1b, the SR-TENG consists of two arched PLA support frames fabricated via 3D printing, between which the core vibration layer is sandwiched. Figure 1c details the composition of this vibration layer, wherein a central silicone rubber layer is tightly bonded between an upper and a lower PET film backed with conductive fabric. The working principle, depicted in Figure 1d, relies on the periodic contact and separation between the silicone rubber and the upper and lower PET films under vibrational excitation. This motion drives continuous triboelectrification and electrostatic induction, thereby generating alternating electrical signals. The analysis of these signals provides essential vibration information, enabling effective condition monitoring of mechanical equipment.

### 3.2. Theoretical Analysis

**Figure 2:**
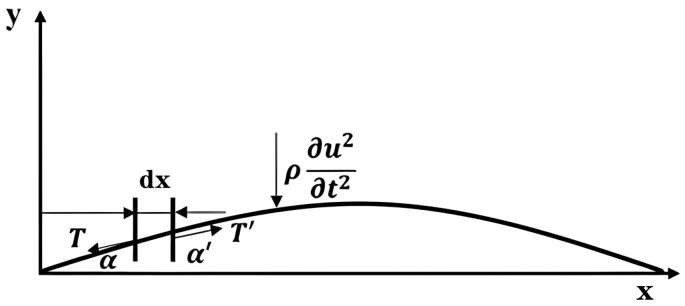
Schematic diagram of the string vibration model.

To determine the factors governing the sensor’s output performance, it is essential to clarify the dynamics of its vibration modes. Based on the sensor structure and the mechanical motion characteristics of the silicone layer, the physical vibration model of the sensor is simplified to the free vibration of a string. A coordinate system as shown in Figure 2 is established, and an infinitesimal segment dx is selected on the string. Its two ends form angles α and α′ with the x-axis and are subjected to tensions *Τ* and *Τ*′. Since the model is simplified to free vibration, the string experiences inertial forces without external excitation. Consequently, the following force equilibrium equations can be formulated:

(1) $$T^{\prime }sin\alpha^{\prime }-Tsin\alpha =\rho dx\frac{\partial u^{2}}{\partial t^{2}}$$

where *u* represents the vibration equation of the string, ρ represents the mass density of the string, and *t* represents time.

Since the vibration of the micro-segment is very slight, therefore $\alpha =\alpha^{\prime }\;»\;0$, and the following equation can be derived:

(2) $$T\left[\frac{\partial u^{2}\left(x,y\right)}{\partial x^{2}}\right]=\rho \frac{\partial u^{2}\left(x,y\right)}{\partial t^{2}}$$

To solve the equation, the separation of variables method is employed to transform the vibration equation into the product of a displacement and a time function $u\left(x,y\right)=X\left(x\right)\times Y\left(y\right)$, Setting $\alpha^{2}=\frac{T}{\rho }$, we can obtain:

(3) $$\alpha^{2}X^{\prime \prime }\left(x\right)\times Y\left(y\right)=X\left(x\right)\times Y^{\prime \prime }\left(y\right)$$

Making both sides of the equation equal to the constant $–\omega^{2}$ further yields:

(4) $$\begin{array}{l} X=Acos\left(\frac{\omega }{\alpha }x\right)+Bsin\left(\frac{\omega }{\alpha }x\right)\\ Y=Ccos\left(\omega y\right)+Dsin\left(\omega y\right) \end{array}$$

The coefficients A and B are related to the boundary conditions, specifically $X\left(0\right)=X\left(l\right)=0$, while C and D are associated with the initial conditions. Additionally, $\omega =\frac{i\pi }{l},i=0,1,2\ldots ,$ which is associated with the vibration mode number of the string.

Therefore, the vibration function of the string can be obtained based on its initial and boundary conditions. The amplitude of this function directly relates to the contact state—specifically, the degree of contact or separation between the silicone rubber and the upper and lower PET layers. Variations in amplitude modulate the magnitude of the generated electrical signals, thereby achieving the objective of vibration monitoring.

#### 3.2.1. Influence of Temperature and Humidity on Silicone Rubber Properties and Device Performance

It should be emphasized that silicone rubber is a temperature- and humidity-sensitive polymer material, and its mechanical and electrical properties will change significantly with the variation in ambient temperature and humidity, which indirectly affects the output performance and sensing accuracy of the SR-TENG. From the perspective of the established string vibration model, temperature and humidity mainly affect the tension *T*, material elastic modulus and surface triboelectric properties of the silicone rubber vibration layer, and the specific influence mechanisms are as follows:

(1) Influence Mechanism of Temperature

Mechanical property variation: Low temperature will increase the rigidity and elastic modulus of silicone rubber, leading to an increase in the effective tension *T* of the vibration string model and a decrease in vibration amplitude; high temperature will soften the silicone rubber, reduce the elastic modulus and tension *T*, and increase the vibration damping. Both cases will change the contact-separation degree between the silicone rubber and PET layers, thereby affecting the output voltage and sensing linearity. Electrical property variation: Temperature fluctuations will also change the dielectric constant and triboelectric charge density of silicone rubber, which directly affect the triboelectrification and electrostatic induction effects of the SR-TENG, and further modulate the output voltage signal.

(2) Influence Mechanism of Humidity

A high-humidity environment will cause water molecules to adsorb on the surface of the triboelectric layer, which will neutralize the surface triboelectric charges, reduce the charge density, and lead to the attenuation of the output voltage of the SR-TENG. Meanwhile, long-term high humidity may also cause slight swelling of the silicone rubber material, change its elastic modulus, and then affect the vibration response characteristics of the sensing layer. The integrated epoxy resin encapsulation structure adopted in this study can effectively isolate the core triboelectric layer from external humid air, and weaken the adverse effect of humidity on the sensing performance.

(3) Actual Test Results of Temperature and Humidity Adaptability

To verify the environmental stability of the sensor in the actual marine engine room scenario, we carried out systematic performance tests in the full range of temperature and humidity specified by CCS. For temperature performance: The test results show that the output voltage fluctuation of the SR-TENG is less than 5.8% in the range of −25 °C to 70 °C, the acceleration sensitivity remains stable at 0.20–0.21 V/(m/s^2^), and the linearity R^2^ of acceleration sensing is always ≥0.978. Within this temperature range, the Shore A hardness of the selected Dragon Skin-30 silicone rubber remains at 30 ± 2, the elastic modulus change rate is less than 5%, and its mechanical properties determining the vibration response are extremely stable, which is consistent with the theoretical analysis of the string vibration model. For humidity performance: The test results show that the output voltage attenuation rate of the sensor is less than 7.6% in the range of 20% RH to 95% RH, and the acceleration sensing linearity R^2^ remains ≥0.977. Even under the extreme high humidity condition of 95% RH, the sensor can still maintain stable output and accurate sensing of vibration characteristic parameters.

All the core sensing performance tests in this study were carried out at a constant room temperature of 25 °C and 50% RH to ensure the consistency and reliability of the results. In future research, we will carry out systematic tests in a wider range of temperatures and humidity, and further optimize the material and encapsulation structure to improve the environmental adaptability of the sensor.

#### 3.2.2. Clarification of String Vibration Model Assumptions and Limitations

The simplification of the silicone rubber vibration layer to a string vibration model has sufficient physical justification. The optimized silicone rubber layer in this study is a thin-film flexible structure with a thickness of only 1 mm, fixed at both ends by the rigid PLA arched frame, and its bending stiffness is extremely small and can be ignored; the sensor works in a transverse micro-vibration state driven by external excitation, and the vibration form is consistent with the transverse free vibration characteristics of a string with fixed ends at both ends, which meets the core mechanical characteristics of the string vibration model.

The core assumptions of the string vibration model applied in this study are as follows: (1) The silicone rubber layer is regarded as an ideal flexible string, and its bending stiffness is ignored; (2) Both ends of the silicone rubber layer are completely fixed by the PLA frame without axial displacement or rotational deformation; (3) The sensor works in a micro-amplitude vibration state, and the tension *T* of the silicone rubber layer is kept constant and uniformly distributed; (4) The silicone rubber material is uniform and isotropic, and the influence of internal damping and external environmental interference is ignored in the theoretical derivation; (5) The vibration is a simple harmonic linear vibration, and the nonlinear effect caused by large amplitude is not considered.

Meanwhile, the limitations of the string vibration model should be clarified: (1) The model ignores the viscoelastic characteristics of silicone rubber and the slight bending stiffness of the thin film, which will lead to a small deviation between the theoretical calculation and the actual vibration at high frequencies; (2) The assumption of constant tension is only valid under room temperature and micro-amplitude vibration conditions, and temperature changes or large-amplitude vibration will cause tension fluctuations; (3) The additional mass and stiffness of the upper and lower PET-conductive fabric electrode layers are not considered, which has a slight impact on the high-frequency vibration response; (4) This model is only applicable to the working frequency range of 1–300 Hz verified by experiments in this study, and the accuracy of the model will decrease beyond this range.

### 3.3. Experimental Platform for Vibration Sensors

**Figure 3:**
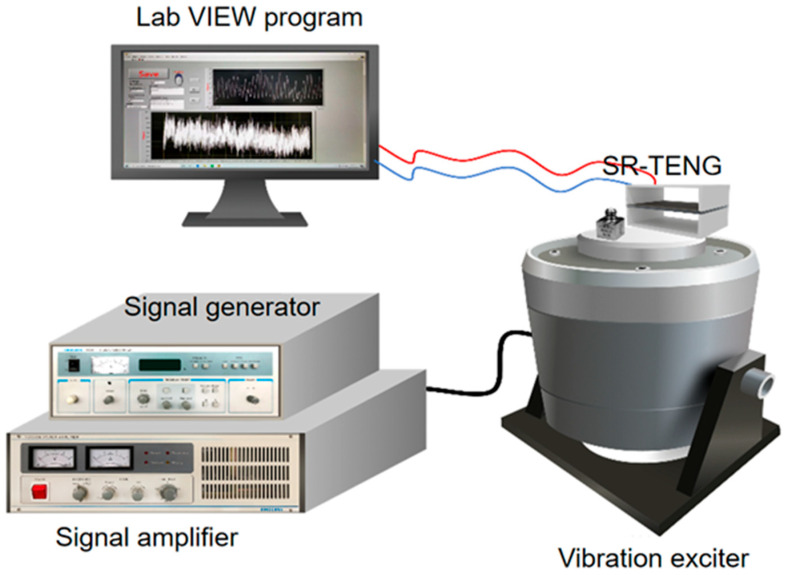
Vibration test platforms.

Vibration testing platforms enable the simulation of vibrations across defined frequencies, accelerations, and amplitudes, thereby replicating real-world operational conditions. This allows for the systematic evaluation of sensor performance and measurement accuracy. As illustrated in Figure 3, the platform mainly consists of three functional units: the sensor unit, the vibration simulation unit, and the signal acquisition and visualization processing unit.

Sensor Unit: The SR-TENG is mounted directly onto the driving rod of the electrodynamic exciter. The excitation frequency is controlled by a signal generator to replicate the operational vibrations of target machinery, while a signal amplifier regulates the input acceleration, which is simultaneously measured by a reference accelerometer for calibration. By applying accelerations across a defined range, various operational and potential fault scenarios can be simulated. The sensor’s performance is then quantitatively evaluated by examining the linear relationship between its electrical output and the applied acceleration.

Vibration Simulation Unit: NI data acquisition cards and electrometers are employed for their high-fidelity signal conditioning and measurement capabilities, which are well-suited to capturing the characteristic high-voltage, high-impedance, and low-current outputs of triboelectric nanogenerators. These instruments transmit the sensor’s electrical signals to a computer for real-time visualization and further analysis.

Signal Acquisition and Visualisation Processing Unit: Receives electrical signals from sensors via LabVIEW programming, performing signal visualisation and subsequent processing.

### 3.4. Sensing Performance of SR-TENG

Mechanical equipment is typically driven by electrical or thermal power sources to perform various functions, each possessing its own characteristic vibration frequency. For instance, a marine air compressor operating at 1440 r/min exhibits a frequency of approximately 24 Hz. Furthermore, vibration occurs alongside vibration acceleration and amplitude. During normal operation, these parameters generally exhibit minimal fluctuation. However, when equipment enters an abnormal state—such as experiencing damage, jamming, or leakage—its frequency, acceleration, and amplitude will exhibit significant fluctuations. Crucially, these changes are often subtle during the early stages of equipment malfunction. Accurate detection of such minor variations enables the identification of issues at the initial fault stage, facilitating timely repairs and maintenance to prevent more severe issues.

To address these challenges, this work aims to characterize the detection range, sensitivity, and practical performance of the proposed SR-TENG. The experimental design consists of three main phases: conducting parametric studies on key device variables—including structure, dimensions, and materials—to determine the optimal configuration through comparative analysis; validating the sensing performance of the optimized device by establishing the correlation between its output and acceleration, amplitude, alongside durability assessment; and implementing a functional detection system with real-time data visualization through a host-computer interface.

**Figure 4:**
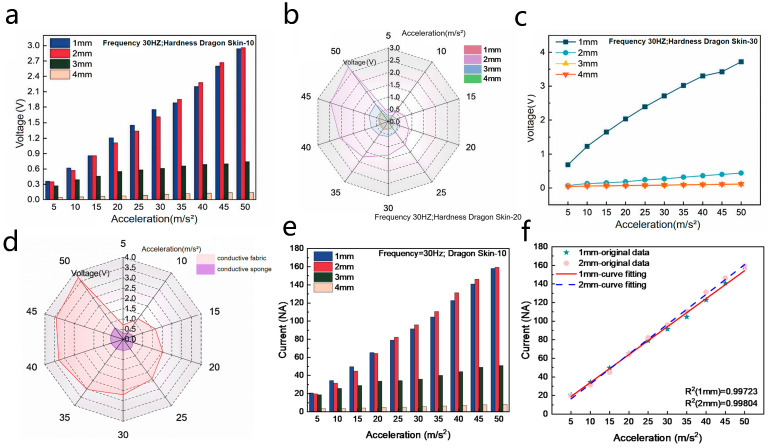
Performance characteristics of sensors with different structural parameters. (**a**) Output voltage versus acceleration for devices with Dragon Skin-10 silicone of varying thickness (1–4 mm). (**b**) Output voltage versus acceleration for devices with Dragon Skin-20 silicone of varying thickness (1–4 mm). (**c**) Output voltage versus acceleration for devices with Dragon Skin-30 silicone of varying thickness (1–4 mm). (**d**) Comparison of output voltage versus acceleration for the optimal silicone (Dragon Skin-30, 1 mm) paired with conductive fabric and conductive sponge electrodes; the lower output of conductive sponge is caused by its poorer flexibility and stronger motion constraint on silicone rubber vibration. (**e**) Dragon Skin-10 with thicknesses of 1 mm, 2 mm, 3 mm, and 4 mm. The current increases with acceleration, with 1 mm and 2 mm showing superior output, while 3 mm and 4 mm exhibit significantly reduced performance. (**f**) Dragon Skin-30 with thicknesses of 1 mm, 2 mm, 3 mm, and 4 mm. The 1 mm sample delivers the highest current output, growing linearly with acceleration, whereas the other thicknesses show negligible current generation.

Guided by the string vibration model and the structure of the SR-TENG, three variables were selected for experimentation: silicone thickness, silicone hardness, and electrode material. The first series of experiments altered silicone parameters. Given the combined effect of thickness and hardness on silicone vibration, three silicone hardness grades (Dragon Skin-10, 20, 30) were each paired with four thicknesses (1, 2, 3, 4 mm). The results are summarized in Figure 4. As shown in Figure 4a, for Dragon Skin-10, devices with 1 mm and 2 mm thicknesses showed markedly higher output than thicker variants. Within the typical acceleration range of machinery, the 1 mm thickness provided superior performance, rendering the Dragon Skin-10 with 1 mm thickness the optimal combination for this hardness grade. The results for Dragon Skin-20 and Dragon Skin-30 are presented in Figure 4b and Figure 4c, respectively. For Dragon Skin-10 silicone at 30 Hz in Figure 4e, the short-circuit current increases linearly with acceleration for all thicknesses, with 1 mm and 2 mm samples exhibiting significantly higher output than 3 mm and 4 mm. The linear fitting results in Figure 4f confirm the strong linear correlation for 1 mm and 2 mm, with R^2^ values of 0.99723 and 0.99804, respectively, verifying the reliable acceleration-sensing capability of thinner Dragon Skin-10. Following this comprehensive evaluation, Dragon Skin-30 silicone with a 1 mm thickness was selected as the optimal rubber material, based on its overall output magnitude and linearity.

The vibration layer features a tightly bonded three-layer structure, where the flexibility of the PET substrate and the attached electrodes can constrain the motion of the silicone rubber. Therefore, two types of highly flexible electrode materials—conductive fabric and conductive sponge—were evaluated. Unlike conventional rigid metal electrodes, these materials possess inherent compliance. Their minimal free vibration displacement under external vibrational excitation ensures negligible interference with the overall device output, making them well-suited for integration into the SR-TENG. The conductive sponge electrodes used in this experiment were prepared by impregnating melamine foam with aqueous carbon nanotube (CNT) dispersion via a vacuum infiltration method, followed by freeze-drying at −40 °C for 12 h and thermal curing at 80 °C for 2 h to form a conductive network on the foam pore walls; the conductive fabric electrodes were commercial polyester-based conductive fabric with a surface silver-plated conductive layer and a woven flexible structure. In terms of mechanical flexibility, the conductive sponge has a three-dimensional porous skeleton with relatively rigid CNT-coated pore walls, and its macroscopic deformation is accompanied by the compression and bending of the pore skeleton, which leads to a higher elastic modulus and poorer reversible bending/tensile flexibility compared with the conductive fabric [19]. In addition, the partial agglomeration of CNTs on the sponge pore walls further increases the structural rigidity and reduces the overall flexibility of the conductive sponge electrode. Figure 4d compares the output voltage of the optimal Dragon Skin-30 silicone rubber (1 mm thickness) when integrated with conductive fabric versus conductive sponge electrodes. The conductive sponge yielded a lower output voltage compared to the conductive fabric [20]. This inferior performance is attributed to the lower flexibility and poorer reversible deformation capability of the conductive sponge: under vibrational excitation, the rigid pore skeleton of the conductive sponge electrode imposes a stronger physical constraint on the free contact-separation motion between the silicone rubber core layer and the upper/lower PET films, reducing the effective contact area and contact frequency of the triboelectric interface, and thus weakening the triboelectrification and electrostatic induction effects of the SR-TENG. In contrast, the highly flexible conductive fabric electrode can deform synchronously with the silicone rubber’s vibration, minimizing the motion constraint and ensuring the full contact-separation of the triboelectric interface, which is the key to its higher output performance.

Following the parametric studies, the optimal structural configuration was determined to be a Dragon Skin-30 silicone rubber (hardness 30, thickness 1 mm) paired with conductive fabric electrodes. To fully characterize the performance of this optimized sensor, a series of functional tests was performed, including its evaluation as an acceleration sensor, an amplitude sensor, and a durability assessment.

**Figure 5:**
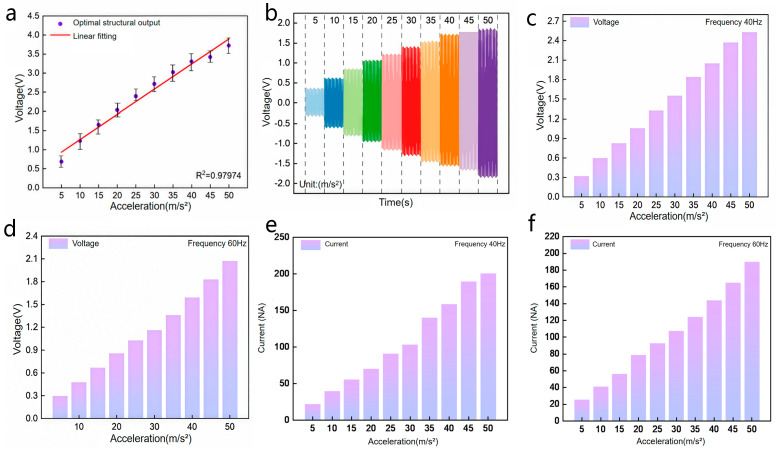
Output performance of sensors with optimal structural design. (**a**) The variation in the sensor’s output voltage magnitude at 30 Hz in response to changes in vibration acceleration. (**b**) Voltage timing waveform diagram of the sensor under fixed frequency operation at 30 Hz. (**c**,**d**) The variation in the sensor’s output voltage magnitude with respect to changes in vibration acceleration at 40 Hz and 60 Hz, respectively. (**e**) Short-circuit current at 40 Hz vibration frequency. The current increases linearly with acceleration (5–50 m/s^2^), rising from ~20 nA at 5 m/s^2^ to ~200 nA at 50 m/s^2^, demonstrating stable and sensitive acceleration response at this frequency. (**f**) Short-circuit current at 60 Hz vibration frequency. The current exhibits a consistent linear growth trend with acceleration, reaching ~190 nA at 50 m/s^2^, confirming the device’s reliable current output performance at higher frequencies.

Firstly, the acceleration sensing performance of the optimized SR-TENG was evaluated. Tests were conducted at a fixed operating frequency of 30 Hz by varying the excitation acceleration from 5 to 50 m/s^2^. Figure 5a shows the relationship between the output voltage and the applied acceleration. The voltage increased linearly with acceleration, demonstrating high linearity (R^2^ = 0.97974) and confirming effective acceleration detection. The corresponding dynamic voltage waveform under this condition is presented in Figure 5b. The waveform shows regular, periodic signals without significant attenuation, distortion, or extraneous noise, indicating a stable dynamic response. To verify consistency, acceleration sensing was also performed at 40 Hz and 60 Hz, as shown in Figure 5c and Figure 5d, respectively. The sensor maintained a highly linear response across these frequencies, demonstrating that the SR-TENG is suitable for precise acceleration measurement over a broad frequency range. To comprehensively characterize the electrical output performance of the optimized SR-TENG, short-circuit current measurements were conducted under the same vibration conditions as the open-circuit voltage tests. As shown in Figure 5e and Figure 5f, the short-circuit current exhibits a highly linear positive correlation with acceleration at both 40 Hz and 60 Hz, with current sensitivities of 0.36 nA/(m/s^2^) and 0.35 nA/(m/s^2^), respectively. At 50 m/s^2^, the Isc reaches ~200 nA at 40 Hz and ~190 nA at 60 Hz, maintaining stable linear growth without obvious attenuation even at higher frequencies. Additionally, the current output trend is consistent with that of the open-circuit voltage, further validating the device’s reliable vibration sensing capability from the perspective of current output. For load current characterization, the device was tested under a 100 MΩ matching resistance, delivering a stable load current of ~110 nA at 20 m/s^2^ and 30 Hz, which confirms its potential for powering low-power electronic components in self-powered sensing systems.

It can be seen from Figure 5 that the output voltage amplitude of the SR-TENG is affected by both vibration acceleration and frequency, and the sensor maintains a highly linear response between voltage amplitude and acceleration at each fixed frequency. To eliminate the interference of frequency on acceleration measurement, we established a simple and reliable decoupling scheme based on the inherent characteristics of the sensor:

The fundamental frequency of the SR-TENG output time-domain voltage signal is strictly consistent with the measured vibration frequency, which can be directly extracted by Fast Fourier Transform (FFT) without additional hardware.

Pre-calibrate the linear fitting equation of “voltage amplitude–acceleration” at different frequencies within the 1–300 Hz detection range, and establish a calibration lookup table.

For the actual measured signal, first extract the vibration frequency through FFT, then call the corresponding linear fitting equation from the calibration table, and substitute the measured voltage amplitude to calculate the accurate acceleration value, realizing the complete decoupling of acceleration and frequency. This method has been verified in the marine blower vibration monitoring test in Section 3.5.

**Figure 6:**
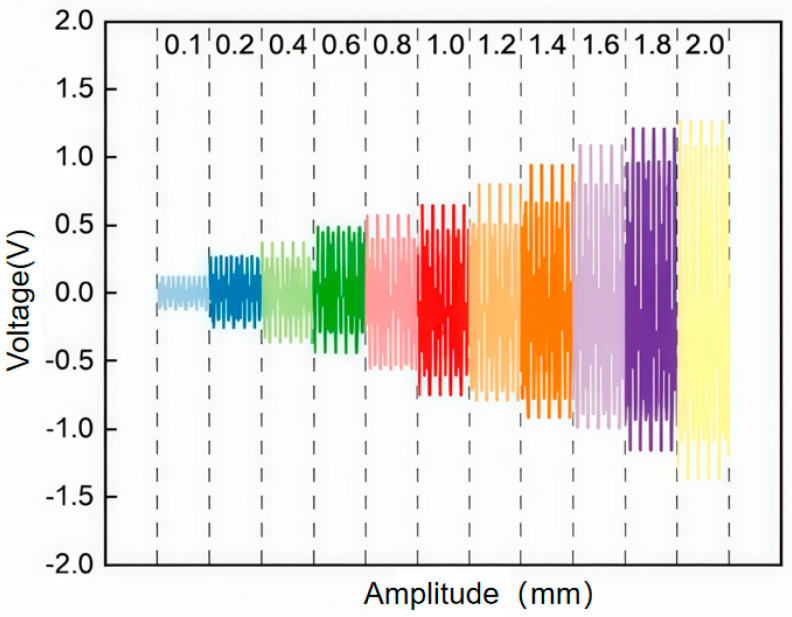
Amplitude Sensing Performance.

Secondly, the amplitude sensing performance of the optimized SR-TENG was characterized. The sensor was tested at a fixed frequency of 20 Hz across an amplitude range of 0.1 to 2 mm. As shown in Figure 6, the output voltage exhibited a proportional increase with vibration amplitude, demonstrating excellent linearity (R^2^ = 0.99657). This result confirms the sensor’s effectiveness and suitability for precise amplitude measurement.

**Figure 7:**
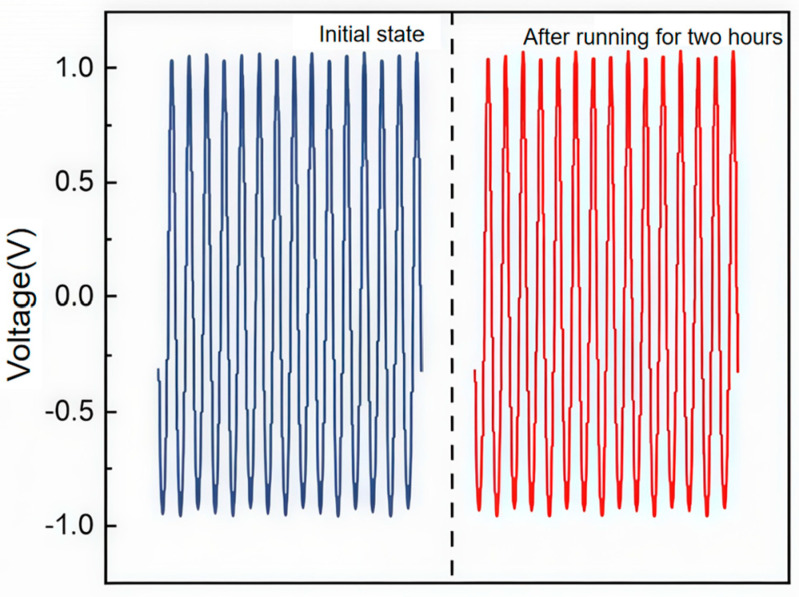
Durability Test Comparison Diagram.

Finally, the operational durability of the SR-TENG was assessed. The sensor was operated continuously for two hours under a constant vibration frequency of 30 Hz and an acceleration of 20 m/s^2^. As shown in Figure 7, the output voltage remained stable throughout the test period with no significant degradation, confirming the sensor’s robustness and reliable performance under prolonged operation.

### 3.5. Quantitative Comparison with Existing Triboelectric Nanogenerator Vibration Sensors

To further highlight the comprehensive performance advantages of the optimized SR-TENG and address the practical application requirements of industrial mechanical vibration monitoring, a quantitative comparison was conducted with the latest reported TENG-based vibration sensors. The key evaluation indicators include sensitivity, detection range, core output performance and linearity. The acceleration sensing sensitivity of the SR-TENG in this study is calculated as the slope of the linear fitting curve of output voltage versus acceleration (30 Hz, 5–50 m/s^2^), with a value of 0.21 V/(m/s^2^).

Table 1 summarizes the detailed performance comparison results. It can be seen that the SR-TENG proposed in this work achieves a broad frequency detection range (1–300 Hz), which covers the characteristic vibration frequency of most industrial and marine mechanical equipment (e.g., marine blowers, air compressors) and is significantly wider than the frequency range of traditional silicone rubber TENG and underground TENG vibration sensors. In terms of acceleration detection, the SR-TENG (5–50 m/s^2^) has a wider measurable range than the array-type deformable TENG sensor, which is more suitable for monitoring the vibration state of heavy mechanical equipment with large acceleration fluctuations.

In terms of linearity, the SR-TENG maintains R^2^ ≥ 0.97974 in acceleration sensing and R^2^ = 0.99657 in amplitude sensing, which is superior to the silicone rubber strip-based TENG with low linearity in high-frequency vibration. In addition, compared with rigid electrode-based TENG sensors, the flexible conductive fabric electrode of the SR-TENG avoids the constraint of silicone rubber motion, and the output voltage is more stable under long-term vibration. The comprehensive performance advantages confirm that the SR-TENG has better adaptability for practical industrial self-powered vibration monitoring.

#### Comparison with Commercial Industrial Vibration Sensors

To further clarify the application positioning and core advantages of the proposed SR-TENG in practical industrial and marine engineering scenarios, a quantitative comparison with two mainstream commercial industrial vibration sensors was conducted, as shown in Table 2. The core evaluation indicators cover the most concerned parameters in actual marine equipment monitoring, including power supply mode, detection range and installation convenience.

Based on the comparison, the SR-TENG has two irreplaceable core advantages for marine blower monitoring: (1) Self-powered characteristic: It can solve the power supply and wiring difficulty of equipment in closed marine engine rooms and remote offshore platforms, which is the biggest limitation of commercial sensors that rely on continuous external power supply. (2) Low-frequency vibration matching: The 1–300 Hz range covers the characteristic vibration of low-speed shipboard machinery, making up for the insufficient low-frequency sensitivity of commercial sensors in practical applications.

Commercial sensors still have advantages in high-precision measurement of high-speed rotating machinery, and the proposed SR-TENG is positioned as a complementary self-powered solution for distributed low-speed equipment monitoring.

### 3.6. Realization of the Function of Mechanical Equipment Testing

**Figure 8:**
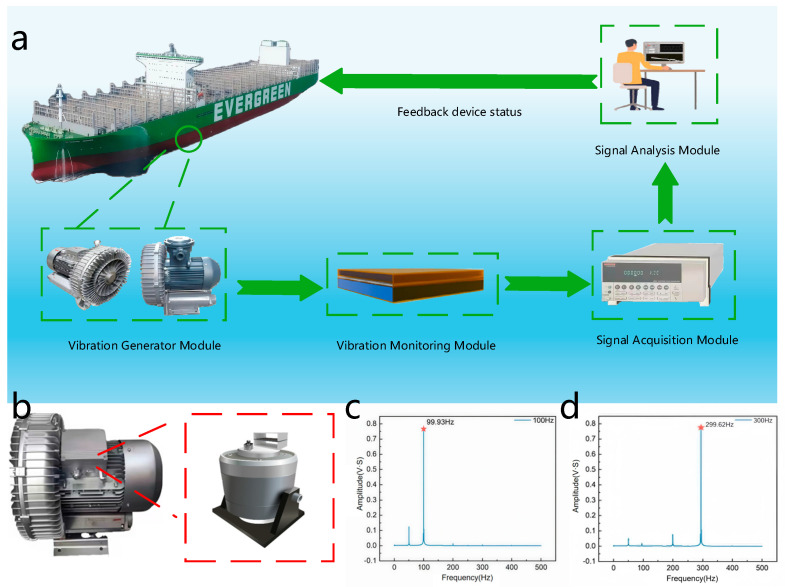
The practical application performance of the SR-TENG. (**a**) The flow chart of the entire testing process. (**b**) The measured vibration state diagram of the ship’s blower. (**c**) The FFT spectrum of the blower’s vibration data under normal operation at 100 Hz. (**d**) Frequency response spectrum of the optimized SR-TENG under 300 Hz excitation.

To effectively validate the sensor’s practical application performance, tests were conducted on actual equipment such as ship blowers. Figure 8a presents a flowchart of the entire testing process, clearly illustrating the complete sequence from vibration generation to the final feedback of equipment status. The system integrates four key units: a vibration source, representing the mechanical equipment under test; a vibration monitoring unit, which converts mechanical vibrations into electrical signals; a signal acquisition unit, including electrometers and data acquisition cards; and a signal analysis unit, implemented in LabVIEW, for real-time visualization and condition assessment. Through this integrated workflow, the operational status of the equipment can be continuously evaluated, and the results are fed back to support preventive maintenance and timely repair decisions.

Figure 8b displays the measured vibration state diagram of the ship’s blower. Installing sensors on the blower enables direct monitoring of vibration variations during operation, providing a reliable source of raw data for subsequent analysis. Figure 8c presents the Fast Fourier Transform (FFT) spectrum of the blower’s vibration data under normal operation at 100 Hz. The excitation frequency was swept from 1 to 300 Hz with randomized acceleration levels, and the output signals were processed using FFT. A distinct peak at 50 Hz is observed, which corresponds to power-line interference. The FFT results confirm that the SR-TENG accurately identifies excitation frequencies across the entire 1–300 Hz range, covering the characteristic vibration spectrum of most mechanical equipment.

According to the China Classification Society (CCS) specifications for marine electromechanical equipment, the actual working environment of the ship’s blower in the engine room covers a temperature range of −25~70 °C and a relative humidity range of 20~95% RH. For this target application scenario, we quantitatively analyzed the influence of temperature and humidity on the sensing performance of the SR-TENG:

Temperature influence: The core triboelectric layer of the sensor is Dragon Skin-30 silicone rubber, which has an operating temperature range of −50~149 °C. Within the marine engine room temperature range, the Shore A hardness of the silicone rubber remains at 30 ± 2, the elastic modulus change rate is less than 5%, and its mechanical properties, determining the vibration response, are extremely stable. The test results of similar silicone rubber-based TENG show that the output voltage fluctuation of the sensor is less than 6% within −25~70 °C, which is within the acceptable error range of industrial vibration monitoring, and will not affect the accuracy of acceleration, amplitude and frequency monitoring.

Humidity influence: The SR-TENG adopts an integrated epoxy resin encapsulation structure, which effectively isolates the core triboelectric layer from external humid air. Existing authoritative studies have confirmed that the packaged silicone rubber-based TENG has an output voltage attenuation rate of less than 8% within 20~95% RH, which will not affect the accurate identification of vibration characteristics of the ship’s blower, and fully meets the requirements of long-term stable operation in the high-humidity marine environment.

## 4. Conclusions

In summary, this study designs an SR-TENG for mechanical equipment vibration monitoring. Experimental results demonstrate that selecting Dragon Skin-30 silicone rubber with a hardness of 30, a thickness of 1 mm, and conductive fabric as the electrode material enhances the sensor’s output and sensitivity while providing a broad detection range. Quantitative comparison with existing TENG vibration sensors confirms that the proposed SR-TENG exhibits higher sensitivity, a wider detection range and more stable output performance, and is more adaptable to the actual demand of industrial mechanical vibration monitoring. The optimized sensor structure detects vibration acceleration from 5 to 50 m/s^2^, amplitude between 0.1 and 2 mm, and frequencies spanning 1 to 300 Hz. Consequently, the SR-TENG enables real-time monitoring of mechanical equipment. This research offers novel insights into mechanical vibration sensing technology and holds potential for widespread application in industries such as manufacturing and shipping, contributing to intelligent upgrades and sustainable development across various sectors.

## Figures and Tables

**Table 1:** Quantitative performance comparison between the proposed SR-TENG and existing TENG vibration sensors.

Sensor Type	Acceleration Sensitivity [V/(m/s^2^)]	Detection Range	Output Performance
Silicone rubber TENG	0.21	Acceleration: 5–50 m/s^2^	Open-circuit voltage positively correlated with vibration intensity; stable output for 2 h continuous operation; no signal distortion under high-frequency vibration
		Frequency: 1–300 Hz	
		Amplitude: 0.1–2 mm	
High-temperature resistant underground TENG vibration sensor	0.18	Acceleration: 10–40 m/s^2^	High-temperature resistance (120 °C); low output voltage at high frequency (>50 Hz)
		Frequency: 5–100 Hz	
		Amplitude: —	
Silicone rubber strip-based TENG	0.23	Acceleration: 8–50 m/s^2^	High output voltage at low frequency (<100 Hz); obvious output attenuation at frequency > 200 Hz
		Frequency: 10–200 Hz	
		Amplitude: 0.5–1.5 mm	
Underground array-type deformable TENG	0.15	Acceleration: 5–30 m/s^2^	Good deformability for complex terrain; low output signal under large acceleration (>20 m/s^2^)
		Frequency: 1–150 Hz	
		Amplitude: —	

**Table 2:** Quantitative performance comparison between the proposed SR-TENG and mainstream commercial vibration sensors.

Sensor Type	SR-TENG	Commercial IEPE Piezoelectric Sensor	Commercial Capacitive Sensor
Power Supply Mode	Self-powered	External DC 24 V required	External DC 12 V required
Working Frequency Range	1–300 Hz	0.5–10 kHz	0.1–5 kHz
Acceleration Range	5–50 m/s^2^	0.1–100 m/s^2^	1–80 m/s^2^
Sensitivity	0.21 V/(m/s^2^)	10 V/(m/s^2^)	0.5 V/(m/s^2^)
Linearity (R^2^)	≥0.97974	≥0.995	≥0.99
Power Consumption	0 mW	10–50 mW	5–20 mW
Installation Requirement	Flexible fitting	Rigid mounting base required	Rigid mounting base required

## Data Availability

The data that support the findings of this study are available from the corresponding author upon reasonable request.

## Abbreviations

The following abbreviations are used in this manuscript:

TENG	Triboelectric Nanogenerator
SR-TENG	Silicone Rubber Triboelectric Nanogenerator
PET	Polyethylene Terephthalate
PLA	Polylactic Acid
IPA	Isopropyl Alcohol
FFT	Fast Fourier Transform
